# Cytomegalovirus Infection and Unusual Early Graft Dysfunction in a Renal Transplant Recipient

**DOI:** 10.4103/0974-777X.52990

**Published:** 2009

**Authors:** Suzan Sanavi, Ahad Ghods, Reza Afshar

**Affiliations:** *Clinical Fellow of Nephrology, Internist, Nephrology Department, Shahed University, Mustafa Khomeini Hospital, Tehran, Iran*; 1 *Professor of Nephrology, Iran University of Medical Sciences, Hashemi-Nejad Medical Center, Tehran, Iran*; 2 *Associate Professor of Nephrology Department, Shahed University, Mustafa Khomeini Hospital, Tehran, Iran*

Sir,

There is an interesting case that may be beneficial for the clinical diagnosis of post transplant complications leading to unusual early graft dysfunction. A 54-year-old woman was referred for a second renal transplantation to our medical center. She was a known case of chronic kidney disease of unknown etiology, who had undergone her first renal transplantation five years ago. Anti-thymocyte globulin (ATG) accompanied with conventional immunosuppressive protocols were administered before the operation,for prevention of rejection. The transplantation operation was performed successfully and proper urine output was established. During the first week post transplant, the patient had no problem and the renal function remained appropriate. At the beginning of the second week she complained of nonproductive cough and malaise without fever. Physical examination and chest radiography were unremarkable. Gradually, the renal function declined progressively, and Doppler sonography and diethylenetriamine penta-acetic acid (DTPA) renal scan revealed acute rejection. Methyl prednisolone pulses were administered and Cytomegalovirus (CMV) antigen-antibodies requested. Because of the positive results of CMV antigen-IgM antibody, intravenous gancyclovir was initiated. The patient responded dramatically to intravenous gancyclovir and eventually, in three weeks, the renal function improved completely. Symptomatic CMV infections typically occur one to six months after transplantation, if prophylaxis is not used, although cases may develop earlier due to more intensive immunosuppressive therapy with Antithymocyte globulin (ATG).[1–3] The most common presentation of CMV disease is a mononucleosis-like syndrome with fever, malaise, myalgias, and arthralgias.[4,5] Interstitial pneumonitis and nephritis are particularly troublesome and cause major morbidity. Renal function may deteriorate in patients with CMV infection, but factors such as decreased renal perfusion, acute tubular necrosis, and transplant rejection may be more important than a direct viral effect on the kidney. There are reports describing the occurrence of CMV-induced transplant glomerulopathy.[6] It has also been suggested that CMV infection is an independent risk factor for the development of rejection.[7] How this might occur is not known, but the net effect is that prevention of CMV infection with intravenous gancyclovir may diminish the incidence of rejection and lead to improved allograft survival.
